# Using period analysis to timely provide serial data on long-term survival for patients with hematological malignancies from Taizhou, Eastern China

**DOI:** 10.3389/fonc.2026.1826643

**Published:** 2026-05-20

**Authors:** Xiaohui Mo, Xin Bing, Lei Hua, Chengyin Xu, Ruijiao Lei, Xukai Chen, Yang Song, Tianhui Chen

**Affiliations:** 1 School of Public Health and Nursing, Hangzhou Normal University, Hangzhou, Zhejiang, China; 2 Department of Cancer Prevention, Zhejiang Cancer Hospital, Hangzhou, Zhejiang, China; 3 Hangzhou Institute of Medicine (HIM), Chinese Academy of Sciences, Hangzhou, Zhejiang, China; 4 Primary Health Care Department, Wuwei Maternal and Child Health Hospital, Wuwei, Gansu, China

**Keywords:** eastern China, hematological malignancies, long-term survival, period analysis, relative survival

## Abstract

**Background:**

While diagnostic and therapeutic innovations have significantly improved the prognosis of hematological malignancies (HMs), comprehensive data on their survival outcomes in China remain scarce. This study aimed to investigate the up-to-date long-term survival of common HMs in a Chinese population.

**Methods:**

In this population-based study, we analyzed data from nine cancer registries in Taizhou, eastern China, covering patients aged ≥15 years diagnosed with HMs between 2009 and 2023. Period analysis was used to estimate overall and stratified 5-, 10-, and 15-year relative survival (RS) by sex, age at diagnosis, region, and subtype, revealing trends in RS over time since diagnosis.

**Results:**

The study included a total of 10, 022 HM cases, with a median age at diagnosis of 62 years. During 2019–2023, the overall 5-year RS for all HMs was 57.0%. Among the three major HM types, lymphoma exhibited the highest 5-year RS (64.4%), followed by multiple myeloma (51.1%), while leukemia had the lowest (49.3%). The 10-year and 15-year RS for all HM patients decreased to 46.0% and 37.5%, respectively. Except for leukemia, females had higher 5-, 10-, and 15-year RS than males. For each type of HM, RS was significantly higher in patients aged<60 years than in those aged ≥60 years, and urban areas showed higher RS than rural areas. In terms of subtypes, chronic lymphocytic leukemia and chronic myeloid leukemia had the best long-term survival. The 15-year survival curves for HMs generally declined with longer time since diagnosis.

**Conclusion:**

This study is the first to provide timely and accurate long-term survival series data for patients with HMs in Taizhou, eastern China, using period analysis. The results offer crucial evidence for optimizing public health strategies and developing targeted cancer control measures.

## 1 Introduction

Hematological malignancies (HMs) constitute a highly heterogeneous group of aggressive diseases, predominantly classified into three major types: leukemia, lymphoma, and multiple myeloma, each with distinct epidemiological patterns, clinical manifestations, treatment regimens, and prognostic features (1). According to GLOBOCAN 2022 statistics, HMs accounted for 1, 309, 970 new cases (6.6% of all cancers) and 699, 461 deaths (7.2% of all cancer-related deaths) globally (2). Non-Hodgkin lymphoma (NHL) was the most prevalent subtype, comprising nearly half of all HM cases. China bears a disproportionately heavy burden of global HMs, with the incidence and mortality burden of NHL, acute lymphocytic leukemia (ALL), and chronic lymphocytic leukemia (CLL) ranking first globally (3). Although HMs are among the most frequently diagnosed cancers in children and adolescents, the majority of cases occur in the elderly population (4). Against the backdrop of a rapidly aging population, the burden of HMs in China—similar to that of many other cancers—is projected to continue increasing (5, 6).

The introduction of novel therapies has substantially improved the overall survival of patients with HMs. Notably, the 5-year relative survival (RS) for Hodgkin lymphoma (HL) and CLL has surpassed 90% in certain developed nations (7). In this context, beyond reporting conventional 5-year RS, it is also essential to focus on long-term survival outcomes extending beyond five years post-diagnosis, as well as the associated long-term health impacts. Presently, the main approaches for evaluating survival include cohort, complete, and period analysis. Period analysis, pioneered by Brenner (8), estimates long-term survival using recent diagnosis data, eliminating the need for complete follow-up data required by traditional cohort analysis (9). This approach offers advantages in both timeliness and accuracy (10). Robust estimation of long-term survival derived from large-scale, high-quality, population-based cancer registry data is essential for providing critical evidence to public health policymakers in formulating and refining cancer prevention and control strategies (11). Zhejiang Province is one of the most developed provinces in eastern China, with a well-established healthcare system. Cancer registration in Zhejiang Province started early, and cancer surveillance data from exemplary cancer registries, such as Xianju County in Taizhou City, have been included several times in “Cancer Incidence in the Five Continents” and CONCORD. Despite this, current research on long-term survival across different HM types in China remains limited, and the application of period analysis in this specific domain is relatively rare.

In this study, we employed period analysis to provide up-to-date series data on 5-, 10-, and 15-year RS estimates for HMs overall and by major types in Taizhou, eastern China, and to reveal trends in long-term cancer survival. We further investigated the impact of sex, age at diagnosis, region, and cancer subtype on patient prognosis. Our findings are expected to provide reliable baseline data and scientific evidence to inform the optimization of clinical practice and the rational allocation of healthcare resources.

## 2 Methods

### 2.1 Data sources

Data on 12, 662 patients diagnosed with HMs between January 1, 2009, and December 31, 2023 (with survival follow-up through December 31, 2023) were obtained from nine cancer registries in Taizhou City, Zhejiang Province. Taizhou covers a population of approximately 6.678 million residents. The analysis focused on three major HM types: lymphoma (including two subtypes: HL and NHL), leukemia (including five subtypes: ALL, acute myeloid leukemia [AML], CLL, chronic myeloid leukemia [CML], and other leukemia), and multiple myeloma. All diagnosed HM cases were coded according to the International Classification of Diseases, 10th Revision (ICD-10) codes (**Supplementary Table S1**). Data were collected from healthcare institutions, health insurance agencies (such as the New Rural Cooperative Medical Scheme and the Urban Resident Basic Medical Insurance), and the Disease Surveillance Points System. A mix of passive and active follow-up methods was used to determine survival status. For passive follow-up, cancer registry staff routinely conducted data linkage with the Hospital Information System (HIS) and the All-Death Surveillance System using identifiable personal information. In addition, registrars actively contacted cancer patients or their families by telephone or community visits to obtain follow-up information. After excluding patients aged<15 years, those lost to follow-up and cases with logical errors identified using IARCcrgTools, 10, 022 eligible cases remained for the final survival analysis.

### 2.2 Statistics analysis

RS is a common method for assessing long-term survival using population-based cancer registry data. RS is defined as the ratio of actual observed survival to expected survival in the general population. Compared to net survival, RS is less susceptible to instability or overestimation associated with older age, prolonged follow-up, or small sample sizes (12). Expected survival was derived from life tables stratified by age, sex, region, and calendar year, using the Ederer II method (13).

Period analysis was employed to estimate 5-, 10-, and 15-year RS for patients diagnosed with HM during 2019–2023, 2014–2023, and 2009–2023, respectively. Period analysis has been shown to provide the most up-to-date survival estimates reflecting contemporary diagnostic and therapeutic standards (14). The analysis used the life table method to estimate RS via the following steps: First, cases were included per the fundamental principles of period analysis. For example, to estimate 5-year RS, the cohort included both patients newly diagnosed in 2019–2023 and pre-2019 diagnosed patients who were alive during 2019–2023. This analysis required standardized handling of left-censored data (diagnoses preceding the study period) and right-censored data (patients alive at the end of the study period). Subsequently, data were structured into a life table to compute the conditional 1-year survival rates (S*_i_*) for the *i* year of follow-up, expressed as:


$${\text{S}}_{i}=1-\frac{{\text{d}}_{i}}{{\text{n}}_{i}-{\text{c}}_{i}/2}$$

In the above equation, n*_i_* was the number of people at the beginning of the *i* year of follow-up, d*_i_* was the number of actual deaths at the end of the *i* year, and c*_i_* was the number of deletions during year *i*.

Finally, the cumulative product of the conditional 1-year survival rates of *k* years yielded the observed survival ( $\bar{S_{k}}$) for year *k*, expressed as:


$$\bar{{\text{S}}_{k}}=\prod_{i=1}^{k}{\text{S}}_{i}$$

RS was the ratio of the observed survival to the expected survival, expressed as:


$${\text{R}}_{i}=\;\frac{\bar{{\text{S}}_{k}}}{{\text{S}}_{k}^{*}}$$

When calculating the 5-year RS, k = 5; similarly, k takes values of 10 and 15 for 10-year and 15-year RS, respectively. In the formula, ${\text{S}}_{k}^{*}$ denoted the expected survival. The complete life table was derived via the Elandt-Johnson model to compute expected survival. RS point estimates and their standard errors (SE) were calculated via Greenwood method. All survival estimates were age-adjusted relative survival using world population standardization for age weights. Data analyses were conducted with the “periodR” package in R software (version 4.3.2), and 15-year RS curves for HMs from 2009 to 2023 were plotted with the “ggplot2” package.

## 3 Results

### 3.1 Basic profile of HMs patients

Overall, 10, 022 records of patients diagnosed with HMs in Taizhou, eastern China between 2009 and 2023 were included in the survival analysis, comprising 4, 848 cases of lymphoma, 3, 620 cases of leukemia, and 1, 554 cases of multiple myeloma (**Table 1**). Median age at diagnosis for all HM patients was 62 years, with males accounting for 58.7% (5, 882 cases) and patients residing in rural areas comprising 74.2% (7, 437 cases). Consistent with the overall distribution of HMs, male patients and those from rural areas accounted for higher proportions across lymphoma, leukemia, and multiple myeloma. Among the three major types, the median age at diagnosis was lowest for leukemia (58 years) and highest for multiple myeloma (67 years). Among lymphoma subtypes, NHL was the predominant subtype during the study period (4, 452 of 4, 848 cases [91.8%]). For leukemia subtypes, AML was the most common (1, 293 of 3, 620 cases [35.7%]), whereas CLL was the least frequent (306 of 3, 620 cases [8.5%]). Detailed distributions are presented for each leukemia and lymphoma subtype, stratified by sex, age at diagnosis, and region (**Supplementary Tables S2**, **S3**).

**Table 1:** Characteristics of patients with hematological malignancies from Taizhou, Eastern China.

Cancer	Characteristics	Diagnostic interval		
		2019–2023 (%)	2014–2023 (%)	2009–2023 (%)
All hematological malignancies	Total	4891 (100)	8491 (100)	10022 (100)
	Sex			
	Male	2808 (57.4)	4982 (58.7)	5882 (58.7)
	Female	2083 (42.6)	3509 (41.3)	4140 (41.3)
	Age at diagnosis (years)			
	<60	1960 (40.1)	3652 (43.0)	4448 (44.4)
	≥60	2931 (59.9)	4839 (57.0)	5574 (55.6)
	Region			
	Urban	1189 (24.3)	2187 (25.8)	2585 (25.8)
	Rural	3702 (75.7)	6304 (74.2)	7437 (74.2)
	Median age at diagnosis (years)	64	62	62
Lymphoma	Total	2377 (100)	4129 (100)	4848 (100)
	Sex			
	Male	1380 (58.1)	2442 (59.1)	2870 (59.2)
	Female	997 (41.9)	1687 (40.9)	1978 (40.8)
	Age at diagnosis (years)			
	<60	965 (40.6)	1792 (43.4)	2149 (44.3)
	≥60	1412 (59.4)	2337 (56.6)	2699 (55.7)
	Region			
	Urban	472 (19.9)	866 (21.0)	1012 (20.9)
	Rural	1905 (80.1)	3263 (79.0)	3836 (79.1)
	Subtype			
	Non-Hodgkin lymphoma	2263 (95.2)	3859 (93.5)	4452 (91.8)
	Hodgkin lymphoma	114 (4.8)	270 (6.5)	396 (8.2)
	Median age at diagnosis (years)	64	62	62
Leukemia	Total	1677 (100)	3005 (100)	3620 (100)
	Sex			
	Male	933 (55.6)	1721 (57.3)	2078 (57.4)
	Female	744 (44.4)	1284 (42.7)	1542 (42.6)
	Age at diagnosis (years)			
	<60	756 (45.1)	1463 (48.7)	1819 (50.2)
	≥60	921 (54.9)	1542 (51.3)	1801 (49.8)
	Region			
	Urban	459 (27.4)	910 (30.3)	1089 (30.1)
	Rural	1218 (72.6)	2095 (69.7)	2531 (69.9)
	Subtype			
	Acute lymphocytic leukemia	173 (10.3)	278 (9.2)	364 (10.1)
	Acute myeloid leukemia	647 (38.6)	1102 (36.7)	1293 (35.7)
	Chronic lymphocytic leukemia	124 (7.4)	254 (8.5)	306 (8.5)
	Chronic myeloid leukemia	236 (14.1)	385 (12.8)	440 (12.2)
	Other leukemia	497 (29.6)	986 (32.8)	1217 (33.5)
	Median age at diagnosis (years)	61	59	58
Multiple myeloma	Total	837 (100)	1357 (100)	1554 (100)
	Sex			
	Male	495 (59.1)	819 (60.4)	934 (60.1)
	Female	342 (40.9)	538 (39.6)	620 (39.9)
	Age at diagnosis (years)			
	<60	197 (23.5)	337 (24.8)	409 (26.3)
	≥60	640 (76.5)	1020 (75.2)	1145 (73.7)
	Region			
	Urban	258 (30.8)	411 (30.3)	484 (31.2)
	Rural	579 (69.2)	946 (69.7)	1070 (68.8)
	Median age at diagnosis (years)	68	67	67

### 3.2 5-year RS for patients with HMs

As shown in **Table 2**, the 5-year RS for all HMs combined in 2019–2023 was 57.0%. Stratified analysis of survival was conducted based on demographic and geographical factors. Stratification by sex revealed a modestly higher 5-year RS in females compared to males (57.9% vs. 56.3%). When stratified by age, a pronounced survival advantage was observed for younger patients (<60 years), whose 5-year RS was 25.6 percentage points higher than that of older patients (≥60 years) (72.5% vs. 46.9%). Additionally, a rural-urban survival gap was noted, with patients in urban areas exhibiting a marginally higher 5-year RS than those in rural areas (57.1% vs. 55.5%).

**Table 2:** The 5-year, 10-year, and 15-year RS of patients with hematological malignancies from Taizhou, eastern China.

Characteristics	2019–2023		2014–2023		2009–2023	
	5-year RS (%)	SE	10-year RS (%)	SE	15-year RS (%)	SE
All hematological malignancies						
Total	57.0	0.8	46.0	0.8	37.5	1.2
Sex						
Male	56.3	1.0	44.1	1.1	35.2	1.5
Female	57.9	1.2	48.7	1.2	40.8	1.8
Age at diagnosis (years)						
<60	72.5	1.0	64.0	1.0	57.7	1.3
≥60	46.9	1.0	33.5	1.1	20.6	2.1
Region						
Urban	57.1	0.9	46.4	1.0	38.6	1.3
Rural	55.5	1.6	43.7	1.5	34.4	2.2
Lymphoma						
Total	64.4	1.1	51.5	1.2	43.6	1.6
Sex						
Male	50.4	1.8	48.2	1.6	40.3	2.0
Female	68.5	1.7	56.5	1.8	48.5	2.4
Age at diagnosis (years)						
<60	80.4	1.3	70.6	1.4	65.6	1.9
≥60	53.7	1.5	38.9	1.6	27.9	2.5
Region						
Urban	65.5	1.3	51.8	1.4	43.8	1.9
Rural	59.5	2.4	48.5	2.3	41.4	2.9
Subtybe						
Non-Hodgkin lymphoma	64.4	1.1	52.0	1.3	44.8	1.7
Hodgkin lymphoma	64.8	4.6	44.7	3.6	34.2	3.5
Leukemia						
Total	49.3	1.3	42.0	1.3	32.1	1.9
Sex						
Male	50.4	1.8	42.1	1.7	32.2	2.3
Female	47.9	2.0	42.0	1.9	32.0	2.8
Age at diagnosis (years)						
<60	65.7	1.7	59.9	1.5	52.8	2.0
≥60	39.3	1.8	30.6	1.7	12.7	3.4
Region						
Urban	51.6	1.6	42.6	1.6	33.9	1.9
Rural	48.0	2.5	40.0	2.2	29.0	3.8
Subtybe						
Acute lymphocytic leukemia	37.7	4.8	30.5	4.1	23.2	3.3
Acute myeloid leukemia	37.4	2.1	32.9	2.1	25.3	3.0
Chronic lymphocytic leukemia	80.9	3.5	61.8	4.4	40.7	8.2
Chronic myeloid leukemia	77.5	3.5	66.9	4.0	59.1	4.3
Other leukemia	45.8	2.3	41.1	2.0	30.1	2.6
Multiple myeloma						
Total	51.1	2.1	37.9	2.1	29.8	3.2
Sex						
Male	50.6	2.8	33.7	2.9	23.2	4.0
Female	51.7	3.1	44.1	3.0	41.1	3.3
Age at diagnosis (years)						
<60	60.9	3.7	48.1	3.6	39.1	4.9
≥60	42.9	2.2	24.4	2.5	13.7	5.0
Region						
Urban	53.2	3.5	42.8	4.4	31.0	2.5
Rural	49.3	2.5	35.8	2.4	23.8	4.0

Among the three major HM types, lymphoma had the highest overall 5-year RS at 64.4%. Subtype analysis showed that the 5-year RS for NHL and HL was nearly identical, at 64.4% and 64.8%, respectively (**Table 2**). Sex-specific analysis revealed a more pronounced survival advantage for female lymphoma patients compared to males (68.5% vs. 50.4%). Younger lymphoma patients had a significantly higher 5-year RS than older patients (80.4% vs. 53.7%), and a regional survival imbalance was also observed (65.5% in urban areas vs. 59.5% in rural areas). Furthermore, similar sex-, age-, and region-related survival patterns to those observed for overall lymphoma were consistently noted for both NHL and HL (**Supplementary Table S4**).

The 5-year RS estimate for multiple myeloma was 51.1%, lower than that for all HMs combined (**Table 2**). A significant survival difference was observed between patients stratified by age at diagnosis, with the 5-year RS decreasing from 60.9% in younger patients to 42.9% in older patients, corresponding to an absolute difference of 18.0%. In contrast, the differences in 5-year RS by sex (51.7% in females vs. 50.6% in males) and by region (53.2% in urban areas vs. 49.3% in rural areas) were relatively modest.

Leukemia exhibited the lowest 5-year RS at 49.3%, but substantial prognostic differences were observed across distinct leukemia subtypes (**Table 2**). Specifically, patients with CLL and CML both had a 5-year RS exceeding 77%, which was more than double that of ALL or AML (approximately 37%). Across overall leukemia and its individual subtypes, younger patients and those residing in urban areas tended to have more favorable survival outcomes. Notably, leukemia displayed a distinct sex-specific survival pattern compared to other HMs, with male patients having a slightly higher 5-year RS than females. Further subgroup analysis of sex-specific differences within leukemia subtypes revealed that female patients had superior survival in ALL and CLL, but poorer outcomes in AML, CML, and other leukemia (**Supplementary Table S5**).

### 3.3 10- and 15-year RS for patients with HMs

Over time since diagnosis, the 10-year RS for all patients with HMs declined to 46.0%, and further decreased to 37.5% by 15 years. Among the three major HM types, the most favorable long-term prognosis was observed for lymphoma, with 10- and 15-year RS of 51.5% and 43.6%, respectively; leukemia followed, with corresponding rates of 42.0% and 32.1%. Notably, although multiple myeloma had a modestly higher 5-year RS than leukemia, its longer-term survival declined more markedly, with 10- and 15-year RS dropping to 37.9% and 29.8%, respectively (**Table 2**).

At the subtype level, NHL consistently demonstrated substantially higher 10- and 15-year RS than HL. Similarly, substantial differences in long-term survival were observed across leukemia subtypes: CML had the best long-term survival, with a 15-year RS as high as 59.1%, whereas ALL showed the poorest, with a 15-year RS of only 23.2%. Stratified analyses based on sex, age at diagnosis, and region revealed that females, patients diagnosed at<60 years, and those in urban areas maintained survival advantages in terms of 10- and 15-year RS across most HMs and major types. An exception was observed for leukemia, where long-term survival was comparable between male and female patients. Stratified data on 10- and 15-year RS for detailed subtypes of lymphoma and leukemia are provided in **Supplementary Tables S4**, **S5**.

### 3.4 15-year relative survival curves for patients with HMs

**Figure 1:**
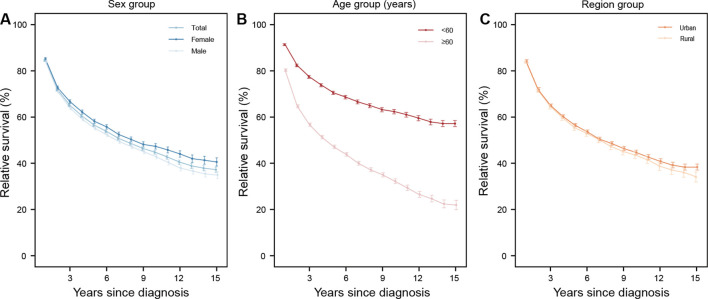
The 15-year relative survival curves for patients with all hematological malignancies in 2009–2023 by sex **(A)**, age **(B)**, and region **(C)** group.

**Figure 2:**
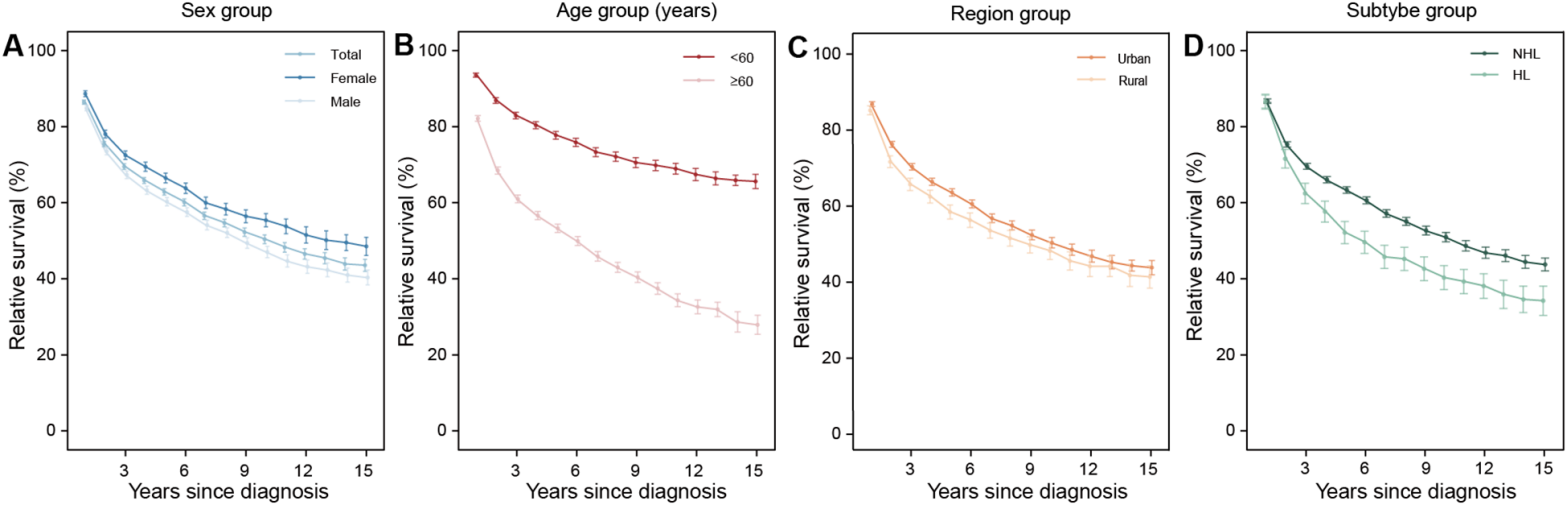
The 15-year relative survival curves for patients with lymphoma in 2009–2023 by sex **(A)**, age **(B)**, region **(C)**, and subtype **(D)** group. NHL, non-Hodgkin lymphoma; HL, Hodgkin lymphoma.

**Figure 3:**
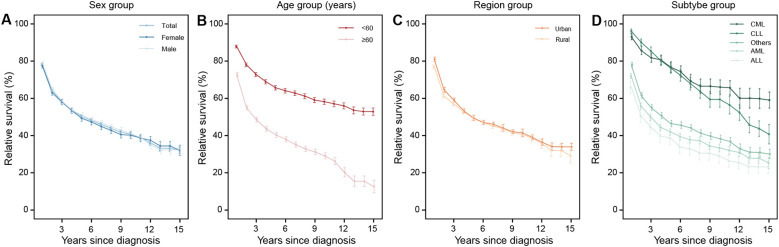
The 15-year relative survival curves for patients with leukemia in 2009–2023 by sex **(A)**, age **(B)**, region **(C)**, and subtype **(D)** group. AML, acute myeloid leukemia; ALL, acute lymphocytic leukemia; CML, chronic myeloid leukemia; CLL, chronic lymphocytic leukemia; Others, other leukemia.

**Figure 4:**
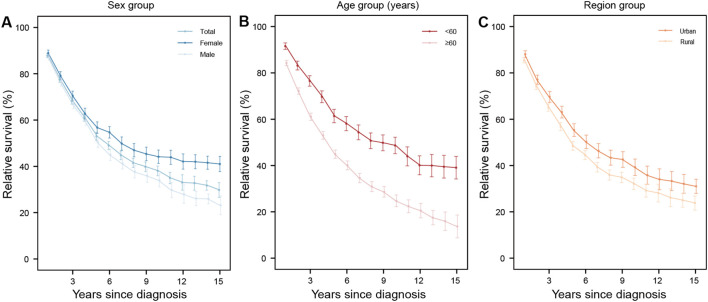
The 15-year relative survival curves for patients with multiple myeloma in 2009–2023 by sex **(A)**, age **(B)**, and region **(C)** group.

**Figures 1**–**4** depict the 15-year survival curves for all HMs and the three major types from 2009 to 2023. In both overall and stratified analyses, the RS of all HM patients declined from 1 to 15 year following diagnosis. Females, patients diagnosed at<60 years, and urban residents consistently demonstrated higher survival throughout the study period (**Figure 1**). These trends were also observed in lymphoma. Furthermore, survival was consistently better for NHL patients than for HL counterparts (**Figure 2**).

For leukemia, survival curves for females and males showed alternating fluctuations in a downward trend over follow-up, with no significant difference between sexes. Survival decline patterns varied by leukemia subtype. CLL showed the most pronounced decline in RS (an approximately linear drop post-diagnosis), while CML declined modestly and gradually stabilized. For the other three subtypes, survival dropped rapidly in the first several years after diagnosis, followed by a slow but persistent decrease (**Figure 3**). A gradual decline in overall RS was also observed for multiple myeloma, though survival curves for younger and female subgroups stabilized after 12 years (**Figure 4**).

Given the considerable heterogeneity in lymphoma and leukemia subtypes across sex, age, and regional subgroups, we further evaluated the 15-year survival trends for each subtype within these populations (**Supplementary Figures S1**, **S2**). Notably, survival curves for leukemia subtypes exhibited marked fluctuations with wide confidence intervals, indicating that reduced sample sizes after further stratification may have increased estimation uncertainty.

## 4 Discussion

This study presents the first comprehensive and timely assessment of long-term survival in patients with HMs in China, utilizing population-based registry data and period analysis. We focused on 5-, 10-, and 15-year RS and performed detailed stratified analyses by sex, age at diagnosis, region, and cancer subtype. The overall 5-year RS for all HMs combined reached 57.0%, with lymphoma showing the highest (64.4%), followed by multiple myeloma (51.1%), and leukemia showing the lowest (49.3%). RS declined significantly from 5 to 15 years post-diagnosis: lymphoma maintained the most favorable long-term prognosis (10-year: 51.5%, 15-year: 43.6%), while multiple myeloma had the poorest (10-year: 37.9%, 15-year: 29.8%). Leukemia subtypes exhibited marked prognostic heterogeneity—CLL and CML had relatively favorable outcomes, whereas ALL and AML had poor survival. Survival patterns varied across subgroups, particularly stratified by age at diagnosis, with patients<60 years showing significantly better survival than those ≥60 years. Urban residents and, in most cases, female patients generally had better long-term prognoses. Notably, 15-year RS curves for almost all HM patients gradually declined over time since diagnosis, with no survival plateau observed, suggesting persistent excess mortality among long-term survivors.

We compared the survival estimates for HM patients in Taizhou with recent national and provincial data. The 5-year RS for lymphoma in Taizhou (2019–2023) was significantly higher than the national (40.8%, 2019–2021) and Zhejiang Provincial (39.6%, 2018–2019) levels. Similarly, the 5-year RS for leukemia was higher than the national (30.6%) and provincial (31.0%) estimates (15, 16). The more optimistic estimates observed in this study are reasonable and may be attributed to several factors. First, unlike the hybrid cohort-period analysis employed in previous studies, our study applied period analysis exclusively, with diagnosis data up to 2023. This approach more accurately reflects the actual improvement in survival resulting from advances in diagnosis and treatment. Second, survival reported for China and Zhejiang—based on diagnosis data up to the end of 2019 and 2017, respectively—are essentially projected estimates and are therefore likely underestimates. Third, Taizhou, as a developed coastal region, has advantages in healthcare accessibility and insurance coverage.

Nevertheless, the survival estimates from our study were lower than those reported in several developed countries. Specifically, the 5-year RS for all HMs combined in Taizhou was lower than the 68.7% reported in Belgium (2014–2018) (17). For major HM types, the 5-year RS for lymphoma was lower than data from many countries in CONCORD-3, where 5-year net survival was approximately 68.0% in the United States, Canada, and Germany (18). The 5-year RS for leukemia was also lower than the corresponding estimates from the United States (67.0% in 2014–2020) and South Korea (55.2% in 2018–2022) (19, 20). For multiple myeloma, the 5-year RS was lower than the rates in the United States (61% in 2014–2020) and Nordic countries (60.0% in 2015–2019), but comparable to South Korea (51.3% in 2018–2022) (7, 19, 20). Although the 5-year RS for CLL (80.9%) was the highest among all HM subtypes in our study, it remained significantly lower than the rates in Nordic countries (90.0% in 2015–2019) and the United States (89.5% in 2012) (7, 21). These population-based differences in cancer survival reflect not only variations in population coverage, time periods, and statistical methodologies across countries or regions, but also differences in factors such as diagnosis precision capacity, access to novel drugs and innovative therapies, implementation of standardized treatments, and allocation of healthcare resources (22). Moving forward, concerted efforts are needed to improve the long-term survival of Chinese patients with HMs through multifaceted strategies, including optimizing healthcare resource allocation, accelerating innovative drug development, promoting multidisciplinary team (MDT) approaches, and advancing personalized treatment.

In China, survival studies of HMs based on cancer registry data have predominantly focused on estimating 5-year survival, while research on longer-term outcomes remains insufficient and notably lags behind. By a comprehensive evaluation of population-based registry data, this study addresses a critical gap in the knowledge of long-term survival for HMs in China. To our knowledge, population-based 10-year survival data in China are limited to Liaoning Province (2000–2002; leukemia: 9.6%; lymphoma: 22.2%) and Qidong City, Jiangsu Province (2012–2016; NHL: 21.5%) (23), with no reports on multiple myeloma. Internationally, existing studies have primarily provided long-term survival estimates for specific HM subtypes. Compared with EUROCARE-6 data (2001–2013) reporting 10-year RS of 55.3% for NHL and 79.3% for HL (24), our estimates were lower (52.0% and 44.7%, respectively). This disparity may be attributed to a higher prevalence of T/NK-cell lymphoma (a more aggressive and relapse-prone subtype) and more frequent diagnosis at advanced stages in Chinese patients compared to Western populations (25, 26), both of which adversely affect initial treatment response and long-term prognosis. Notably, our study observed certain leukemia subtypes with comparable or superior survival outcomes in international comparisons. The 10-year RS for CLL (61.8%), CML (66.9%), and AML (32.9%) in Taizhou exceeded European estimates (56.8%, 52.5%, and 15.6%) (24, 27). Similarly, survival for most leukemia subtypes (except CLL) was generally higher than the corresponding estimates from Germany (2012–2016) (28). This may be related to the younger age profile of leukemia patients in Taizhou and a lower incidence of poor-prognosis genetic mutations in China (29). Furthermore, this advantage is likely supported by advances in diagnostic technologies in China, particularly achieving 100% coverage of morphological characteristics, immunophenotype, cytogenetics and molecular biology testing by 2020 (30). However, these differences should be interpreted cautiously due to non-overlapping comparison periods and the limited cohort size, which constrain statistical power.

Over recent decades, therapeutic advances in HMs have been at the forefront of cancer clinical research, with greater survival improvements than other common cancers (31, 32). As survival extends, treatment-related late complications have correspondingly increased, posing a major challenge in the long-term management of HMs. For most HM subtypes—even those like lymphoma, typically considered highly curable—survival curves continue to decline years after diagnosis, without reaching a plateau. This persistent mortality risk stems from several factors. First, late disease relapse can occur. For instance, a study of 162 patients with diffuse large B-cell lymphoma showed a 19% recurrence rate after more than five years of sustained complete remission (33). Second, long-term survivors face risks of one or more late effects due to acute or delayed toxicities from prior treatments such as alkylating chemotherapy or mediastinal radiotherapy, including secondary cancers, cardiovascular diseases, and other conditions (34). These late mortality risks remain elevated even 20 years or more after treatment (35, 36).

In this context, the outcomes for CML are relatively favorable. Patients with CML exhibited the best 10- and 15-year RS among all HM subtypes, with survival curves gradually stabilizing. This favorable outcome is largely attributable to the widespread use of tyrosine kinase inhibitors (TKIs) and hematopoietic stem cell transplantation (HSCT), which have enabled most patients to achieve a life expectancy approaching that of the general population (37). Nevertheless, a significant proportion of long-term survivors remain uncured even 15 years after diagnosis, underscoring the need for management strategies to enhance surveillance for late relapse alongside improving short-term outcomes. Concurrently, actively screening for and managing long-term treatment-related complications is essential to improve long-term quality of life for cancer survivors.

For most patients with HMs, females generally experience a better long-term prognosis. The reasons for this sex-based disparity remain unclear but may involve sex hormone-mediated biological advantages and differences in health-related behaviors (38, 39). Notably, male leukemia patients in this study had comparable or even superior survival compared to females, potentially indicating that certain current treatment regimens may benefit males more, though random variation due to limited case numbers cannot be excluded (40). Consistent with other population-based studies, older patient groups generally face a poorer prognosis than younger patients (28). This may be attributed to diminished organ compensatory reserve capacity and a higher comorbidity burden in elderly patients, which can accelerate mortality (41). Furthermore, elderly patients often exhibit poorer tolerance for curative-intensive chemotherapy, frequently necessitating dose reductions or selection of milder regimens, which limits optimal survival benefit (42). Our analysis also revealed superior long-term survival in urban versus rural patients, with this gap stemming from lower medical care standards and limited healthcare resources in rural areas (43). Rural patients typically have lower incomes, less education, and a higher likelihood of late-stage diagnosis than urban counterparts. These social determinants contribute to their inferior survival outcomes (44). Therefore, establishing a more equitable healthcare system and developing targeted interventions for vulnerable populations are urgently needed. Such comprehensive efforts can narrow the survival gap and enhance the overall control and prevention of HMs in China.

Our study has several strengths and limitations. First, this is the first systematic report of updated long-term survival series data for all HM patients and the three main types in Eastern China, covering 5-, 10-, and 15-year RS. Second, period analysis better reflects patient prognosis under current diagnostic and therapeutic standards, offering greater timeliness and accuracy than traditional methods such as the cohort approach. Third, the use of population-based cancer registries minimizes the selection bias inherent in hospital-based studies. However, as a population-based study, it lacks detailed information on treatment regimens and other clinical parameters, such as disease stage or cytogenetic profiles, which precludes analysis of their impact on survival. Furthermore, as the data originate solely from Eastern China, future studies with provincial or national data are needed for more comprehensive analyses.

## 5 Conclusion

In summary, this study provides the first timely and accurate long-term survival estimates for HM patients in Taizhou, Eastern China, using period analysis. Although a favorable 5-year RS was observed, 10- and 15-year RS were significantly lower, highlighting the need to improve long-term prognosis. The study also revealed survival disparities across multiple dimensions, including sex, age at diagnosis, region, and cancer subtype, which aids in identifying vulnerable patient subgroups. These findings establish a crucial baseline for local cancer surveillance and provide essential evidence for optimizing long-term follow-up strategies, rationally allocating healthcare resources, and developing targeted cancer control measures.

## Data Availability

The original contributions presented in the study are included in the article/**Supplementary Material**, further inquiries can be directed to the corresponding author.
