# Supplementary material for: Using period analysis to timely provide serial data on long-term survival for patients with hematological malignancies from Taizhou, Eastern China
Source: Front Oncol. 2026 May 20;16:1826643. doi: 10.3389/fonc.2026.1826643 (PMC13229762; doi:10.3389/fonc.2026.1826643)
Supplement: Supplementary file 1 [file DataSheet1.pdf]

Supplementary Table S1. ICD-10 of investigated hematological malignancies.

Hematological malignancies	ICD-10
**Leukemia**	C91–C93.7, C93.9–C95.2, C95.7–C95.92, Z80.6, Z85.6
Acute lymphoid leukemia	C91.0–C91.02, C91.2–C91.32, C91.6–C91.62
Acute myeloid leukemia	C92.0–C92.02, C92.3–C92.62, C93.0–C93.02, C94.0, C94.02, C94.2–C94.22, C94.4–C94.5
Chronic lymphoid leukemia	C91.1–C91.12
Chronic myeloid leukemia	C92.1–C92.22
Other leukemia	C91.2–C91.9, C92.2, C92.7–C92.9, C93.1–C93.9, C94.1, C94.3, C94.6–C95.9
**Multiple myeloma**	C88–C90.9
**Lymphoma**	C81–85, C96
Non-Hodgkin lymphoma	C82–C86.6, C96–C96.9
Hodgkin lymphoma	C81–C81.49, C81.7–C81.79, C81.9–C81.99, Z85.71–Z85.72

Supplementary Table S2. Characteristics of patients with lymphoma from Taizhou, Eastern China.

Cancer	Characteristics	Diagnostic interval		
		2019–2023 (%)	2014–2023 (%)	2009–2023 (%)
Non-Hodgkin lymphoma	Total	2263 (100)	3859 (100)	4452 (100)
	Sex			
	Male	1313 (58.0)	2275 (59.0)	2630 (59.1)
	Female	950 (42.0)	1584 (41.0)	1822 (40.9)
	Age at diagnosis (years)			
	<60	896 (39.6)	1650 (42.8)	1945 (43.7)
	≥60	1367 (60.4)	2209 (57.2)	2507 (56.3)
	Region			
	Urban	442 (19.5)	795 (20.6)	911 (20.5)
	Rural	1821 (80.5)	3064 (79.4)	3541 (79.5)
	Median age at diagnosis (years)	64	63	62
Hodgkin lymphoma	Total	114 (100)	270 (100)	396 (100)
	Sex			
	Male	67 (58.8)	167 (61.9)	240 (60.6)
	Female	47 (41.2)	103 (38.1)	156 (39.4)
	Age at diagnosis (years)			
	<60	69 (60.5)	142 (52.6)	204 (51.5)
	≥60	45 (39.5)	128 (47.4)	192 (48.5)
	Region			
	Urban	30 (26.3)	71 (26.3)	101 (25.5)
	Rural	84 (73.7)	199 (73.7)	295 (74.5)
	Median age at diagnosis (years)	55	58	58

Supplementary Table S3. Characteristics of patients with leukemia from Taizhou, Eastern China.

Cancer	Characteristics	Diagnostic interval		
		2019–2023 (%)	2014–2023 (%)	2009–2023 (%)
Acute lymphocytic leukemia	Total	173 (100)	278 (100)	364 (100)
	Sex			
	Male	89 (51.4)	147 (52.9)	201 (55.2)
	Female	84 (48.6)	131 (47.1)	163 (44.8)
	Age at diagnosis (years)			
	<60	82 (47.4)	136 (48.9)	186 (51.1)
	≥60	91 (52.6)	142 (51.1)	178 (48.9)
	Region			
	Urban	51 (29.5)	85 (30.6)	110 (30.2)
	Rural	122 (70.5)	193 (69.4)	254 (69.8)
	Median age at diagnosis (years)	50	50	49
Acute myeloid leukemia	Total	647 (100)	1102 (100)	1293 (100)
	Sex			
	Male	360 (55.6)	606 (55.0)	697 (53.9)
	Female	287 (44.4)	496 (45.0)	596 (46.1)
	Age at diagnosis (years)			
	<60	288 (44.5)	547 (49.6)	659 (51.0)
	≥60	359 (55.5)	555 (50.4)	634 (49.0)
	Region			
	Urban	167 (25.8)	299 (27.1)	349 (27.0)
	Rural	480 (74.2)	803 (72.9)	944 (73.0)
	Median age at diagnosis (years)	62	60	59
Chronic lymphocytic leukemia	Total	124 (100)	254 (100)	306 (100)
	Sex			
	Male	70 (56.5)	158 (62.2)	190 (62.1)
	Female	54 (43.5)	96 (37.8)	116 (37.9)
	Age at diagnosis (years)			
	<60	46 (37.1)	100 (39.4)	127 (41.5)
	≥60	78 (62.9)	154 (60.6)	179 (58.5)
	Region			
	Urban	41 (33.1)	78 (30.7)	105 (34.3)
	Rural	83 (66.9)	176 (69.3)	201 (65.7)
	Median age at diagnosis (years)	64	63	63
Chronic myeloid leukemia	Total	236 (100)	385 (100)	440 (100)
	Sex			
	Male	141 (59.7)	235 (61.0)	272 (61.8)
	Female	95 (40.3)	150 (39.0)	168 (38.2)
	Age at diagnosis (years)			
	<60	136 (57.6)	237 (61.6)	271 (61.6)
	≥60	100 (42.4)	148 (38.4)	169 (38.4)
	Region			
	Urban	58 (24.6)	92 (23.9)	103 (23.4)
	Rural	178 (75.4)	293 (76.1)	337 (76.6)
	Median age at diagnosis (years)	56	55	54
Other leukemia	Total	497 (100)	986 (100)	1217 (100)
	Sex			
	Male	273 (54.9)	575 (58.3)	718 (59.0)
	Female	224 (45.1)	411 (41.7)	499 (41.0)
	Age at diagnosis (years)			
	<60	204 (41.0)	443 (44.9)	576 (47.3)
	≥60	293 (59.0)	543 (55.1)	641 (52.7)
	Region			
	Urban	142 (28.6)	356 (36.1)	422 (34.7)
	Rural	355 (71.4)	630 (63.9)	795 (65.3)
	Median age at diagnosis (years)	65	61	61

Supplementary Table S4. The 5-year, 10-year, and 15-year RS of patients with lymphoma from Taizhou, eastern China.

Characteristics	2019–2023		2014–2023		2009–2023	
	5-year RS（%）	SE	10-year RS（%）	SE	15-year RS（%）	SE
Non-Hodgkin lymphoma						
Sex						
Male	61.7	1.5	48.6	1.7	40.5	2.2
Female	68.2	1.7	57.2	1.9	52.4	2.3
Age at diagnosis (years)						
<60	80.8	1.4	70.6	1.5	65.1	2.1
≥60	54.5	1.5	40.2	1.7	30.3	2.6
Region						
Urban	65.2	1.3	54.1	2.1	45.4	1.9
Rural	61.6	2.4	49.2	3.1	41.3	3.5
Hodgkin lymphoma						
Sex						
Male	60.9	5.9	41.5	4.4	33.6	3.9
Female	73.2	6.8	52.0	5.8	36.6	5.8
Age at diagnosis (years)						
<60	85.9	4.1	75.4	3.9	47.4	4.3
≥60	49.1	5.0	26.1	4.8	19.6	5.7
Region						
Urban	70.1	4.2	49.3	4.1	37.5	4.2
Rural	53.7	4.9	36.5	5.0	31.0	4.9

Supplementary Table S5. The 5-year, 10-year, and 15-year RS of patients with leukemia from Taizhou, eastern China.

Characteristics	2019–2023		2014–2023		2009–2023	
	5-year RS（%）	SE	10-year RS（%）	SE	15-year RS（%）	SE
Acute lymphocytic leukemia						
Sex						
Male	32.7	4.3	29.2	5.1	22.7	3.7
Female	46.2	4.2	31.2	4.2	26.0	4.6
Age at diagnosis (years)						
<60	63.8	4.5	52.3	4.1	35.2	4.6
≥60	35.3	4.5	29.0	3.1	22.1	4.0
Region						
Urban	49.2	4.8	36.6	4.9	27.0	4.2
Rural	28.9	5.2	25.4	4.2	18.2	3.2
Acute myeloid leukemia						
Sex						
Male	38.5	3.1	31.0	3.1	20.8	4.6
Female	35.6	3.0	33.8	2.7	27.7	2.4
Age at diagnosis (years)						
<60	60.9	2.9	55.4	2.7	50.6	3.6
≥60	26.0	2.6	20.2	2.5	14.1	3.6
Region						
Urban	37.9	4.0	33.9	3.6	29.8	2.4
Rural	35.5	2.5	30.7	2.5	18.1	4.2
Chronic lymphocytic leukemia						
Sex						
Male	76.0	4.8	61.4	4.0	46.2	3.8
Female	89.0	4.2	62.7	4.8	34.2	5.0
Age at diagnosis (years)						
<60	83.2	4.2	72.1	4.5	55.9	5.1
≥60	77.6	4.8	53.1	4.2	25.1	4.5
Region						
Urban	84.3	4.2	69.1	4.8	47.9	4.6
Rural	73.6	4.3	56.3	3.9	39.5	4.5
Chronic myeloid leukemia						
Sex						
Male	79.2	4.4	67.1	4.0	59.7	4.3
Female	74.4	4.5	64.2	4.4	57.7	4.2
Age at diagnosis (years)						
<60	91.5	2.5	79.9	4.1	74.0	4.0
≥60	69.4	4.3	59.5	4.8	43.1	4.4
Region						
Urban	84.7	4.2	67.8	4.5	60.5	4.1
Rural	75.0	4.3	66.5	4.6	53.3	4.5
Other leukemia						
Sex						
Male	47.9	3.0	39.5	2.6	27.1	3.2
Female	42.5	3.5	43.4	3.1	33.1	3.4
Age at diagnosis (years)						
<60	58.7	3.2	46.2	2.6	34.6	2.9
≥60	37.9	2.8	27.9	2.7	14.4	3.9
Region						
Urban	47.0	2.8	44.2	2.5	30.9	2.7
Rural	42.5	4.1	36.0	3.3	28.1	4.5

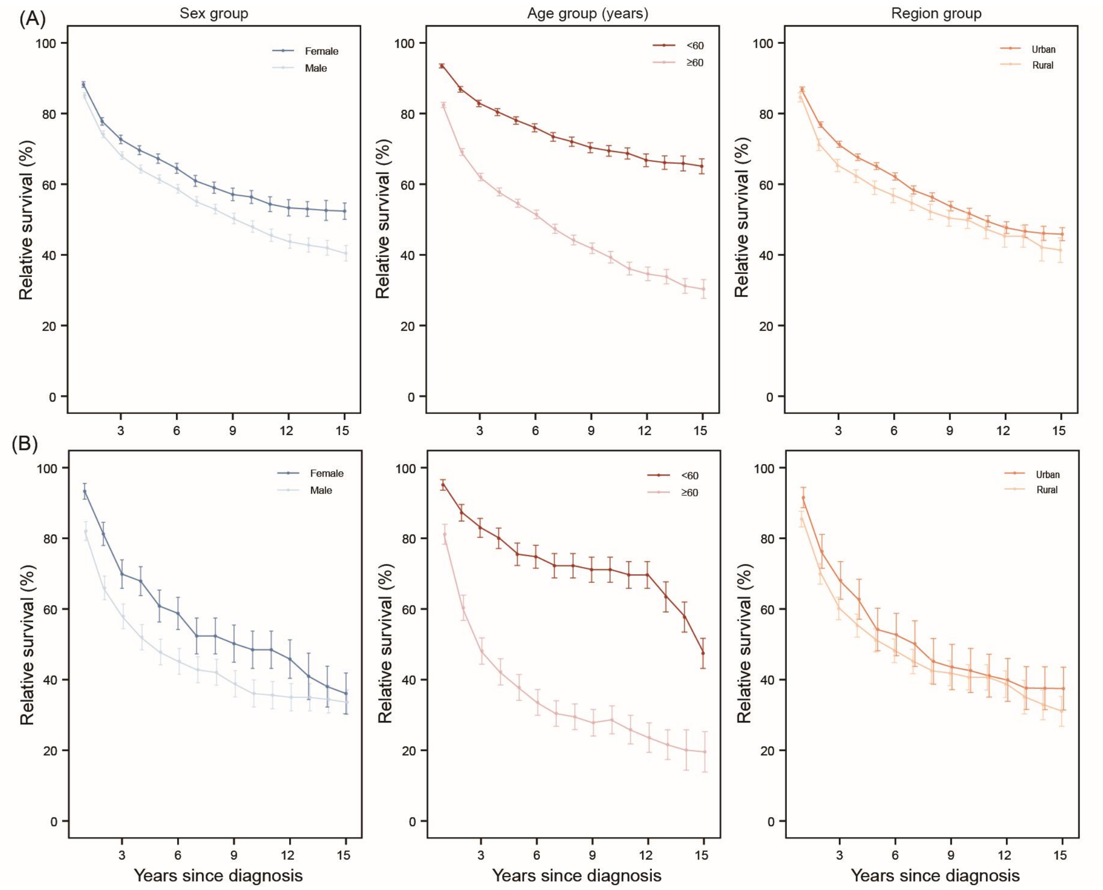

Supplementary Figure S1. Trends of 15-year relative survival for lymphoma patients during 2009–2023 by sex, age at diagnosis, and region. (A) Non-Hodgkin lymphoma; (B) Hodgkin lymphoma.

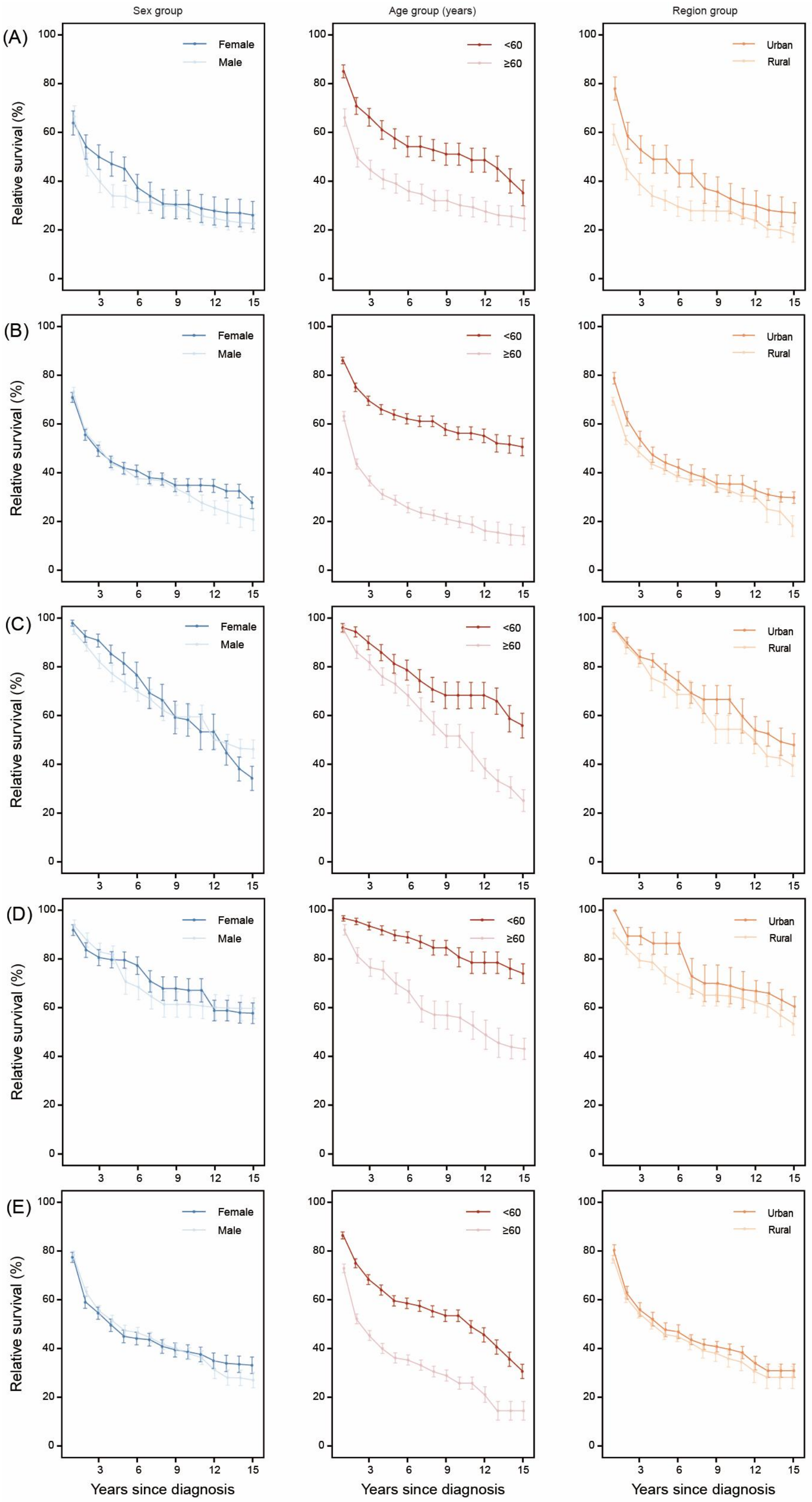

Supplementary Figure S2. Trends of 15-year relative survival for leukemia patients during 2009–2023 by sex, age at diagnosis, and region. (A) Acute lymphocytic leukemia; (B) Acute myeloid leukemia; (C) Chronic lymphocytic leukemia; (D) Chronic myeloid leukemia; (E) Other leukemia.
